# Proteomic Analysis of the Senescence-Associated Secretory Phenotype: GDF-15, IGFBP-2, and Cystatin-C Are Associated With Multiple Aging Traits

**DOI:** 10.1093/gerona/glad265

**Published:** 2023-11-20

**Authors:** Daniel S Evans, Danielle Young, Toshiko Tanaka, Nathan Basisty, Stefania Bandinelli, Luigi Ferrucci, Judith Campisi, Birgit Schilling

**Affiliations:** California Pacific Medical Center Research Institute, San Francisco, California, USA; Department of Epidemiology and Biostatistics, University of California San Francisco, San Francisco, California, USA; California Pacific Medical Center Research Institute, San Francisco, California, USA; Department of Epidemiology and Biostatistics, University of California San Francisco, San Francisco, California, USA; Longitudinal Studies Section, Translational Gerontology Branch, NIA, NIH, Baltimore, Maryland, USA; Longitudinal Studies Section, Translational Gerontology Branch, NIA, NIH, Baltimore, Maryland, USA; Geriatric Unit, Azienda Sanitaria Firenze (ASF), Florence, Tuscany, Italy; Longitudinal Studies Section, Translational Gerontology Branch, NIA, NIH, Baltimore, Maryland, USA; Buck Institute for Research on Aging, Novato, California, USA; Buck Institute for Research on Aging, Novato, California, USA; (Biological Sciences Section)

**Keywords:** Aging biomarkers, Cellular senescence, Gait speed, Grip strength, Proteomics

## Abstract

Cellular senescence, a hallmark of aging, results in a senescence-associated secretory phenotype (SASP) with an increased production of proinflammatory cytokines, growth factors, and proteases. Evidence from nonhuman models demonstrates that SASP contributes to tissue dysfunction and pathological effects of aging. However, there are relatively few human studies on the relationship between SASP and aging-related health outcomes. Proteins from the SASP Atlas were measured in plasma using aptamer-based proteomics (SomaLogic). Regression models were used to identify SASP protein associations with aging-related traits representing multiple aspects of physiology in 1 201 participants from 2 human cohort studies (BLSA/GESTALT and InCHIANTI). Traits examined were fasting glucose, C-reactive protein, interleukin-6, alkaline phosphatase, blood urea nitrogen, albumin, red blood cell distribution width, waist circumference, systolic and diastolic blood pressure, gait speed, and grip strength. Study results were combined with a fixed-effect inverse-variance weighted meta-analysis. In the meta-analysis, 28 of 77 SASP proteins were significantly associated with age. Of the 28 age-associated SASP proteins, 18 were significantly associated with 1 or more clinical traits, and 7 SASP proteins were significantly associated with 3 or more traits. Growth/differentiation factor 15, Insulin-like growth factor-binding protein 2, and Cystatin-C showed significant associations with inflammatory markers and measures of physical function (grip strength or gait speed). These results support the relevance of SASP proteins to human aging, identify specific traits that are potentially affected by SASP, and prioritize specific SASP proteins for their utility as biomarkers of human aging.

Human life expectancy has steadily increased over the last century, and the proportion of the population aged 65 years and older has increased in many developed countries (1,2). Advanced age is recognized as a major risk factor for virtually all major causes of death, as well as multimorbidity and disability (3–5). Efforts to lengthen healthspan have focused on compressing the years spent in poor health and morbidity at the end of life (6).

Recognizing that age is a major risk factor for late-life morbidity, the geroscience hypothesis states that targeting fundamental aging mechanisms could reduce the incidence of multiple age-related diseases and extend healthspan (7). Cellular senescence is one of the hallmarks of aging (8,9), and the number of senescent cells increases with age in mouse models (10,11). Senescence can be triggered by a variety of cellular stressors, including DNA damage, oncogene activation, and mitochondrial dysfunction (11). Senescent cells are viable but their cell cycles are arrested, they can be resistant to apoptosis, and they typically release bioactive compounds into the extracellular space which is cumulatively known as the senescence-associated secretory phenotype (SASP) (11). SASP components include proteins such as cytokines, chemokines, growth factors, and proteases that can have proinflammatory, proapoptotic, and profibrotic effects (11–13).

Compelling evidence from experiments in non-human models supports the notion that cellular senescence and the SASP can contribute to a wide range of aging pathologies. Experiments in animal models that manipulated the number of senescent cells provided evidence that cellular senescence is associated with multiple age-related pathologies and conditions, including age-related bone loss, physical function and lifespan, metabolic and adipose tissue dysfunction, atherosclerosis, and osteoarthritis (11). Senescence associations with multiple age-related conditions in non-human models have sparked a growing interest in SASP as a target for therapeutics (11). Evaluating the relationship between SASP proteins and a diverse set of human aging traits in large clinical studies would provide critical information that can lay the groundwork for future intervention studies using senolytics to test the geroscience hypothesis. Furthermore, identifying specific SASP proteins that may be associated with different aspects of clinical aging and physiology could aid in the development of precise diagnostic and therapeutic approaches to target SASP for human aging.

Recent emerging evidence from studies measuring selected SASP factors suggests that SASP factors are associated with selected aging-related traits in humans, but a comprehensive survey of SASP factor associations with a wide range of human clinical outcomes has not been conducted. A study of 32 SASP circulating proteins in 76 postmenopausal women identified significant SASP protein associations with age and 2 reproductive aging measures (age at menopause and years since menopause) (14). An examination of 24 circulating SASP proteins found significant associations with post-surgical outcomes and a frailty index built from comorbidity counts and activities of daily living among 115 biobank individuals, 97 older adults undergoing surgery for severe aortic stenosis, and a case-control study of older women undergoing ovarian cancer surgery (18 cases, 18 controls) (15). An index of SASP proteins was associated with antidepressant response to late-life depression in a nonrandomized trial of 416 older adults participants with major depressive disorder (16), but the SASP index used by Diniz et al. did not include many of the SASP proteins that have been identified, including GDF-15 (13). In a recent study of 1 377 LIFE study participants, 27 SASP proteins were associated with physical function, as assessed using the short physical performance battery and its subcomponents and 400-meter walk time (17). Proteomic studies of aging found that circulating proteins associated with human chronological age are enriched for SASP proteins (18), and a proteomic age signature that includes SASP proteins was associated with chronic diseases and mortality (19). However, many SASP proteins were not components of the proteomic age signature, and the extent to which each SASP protein is associated with age-related traits remains unknown (19). Although published results point to the importance of SASP in human aging, individual SASP protein associations have been examined for only a limited number of aging outcomes, or SASP components were combined into an index that prevented the identification of individual SASP factor associations. With the evidence from non-human model organisms that senescence and SASP can be related to a wide array of diseases, there is a need to evaluate whether SASP proteins are also associated with multiple aspects of aging in humans.

In this report, we sought to identify which proteins from the SASP Atlas were associated with age and age-related clinical traits using proteomics from 1 201 participants from 2 geographically distinct population-based studies of aging: BLSA/GESTALT (USA) and InCHIANTI (Italy). Participants in these studies were well-characterized, allowing for the assessment of health across a wide range of physiological functions and adjustment for important confounding factors. Aptamer-based proteomic workflows were used to capture 77 proteins from a core set of SASP proteins from the SASP Atlas that were previously described to be elevated by 3 senescence inducers (13). SASP proteins that were found to be associated with age were then tested for association with multiple aging-related traits in the following categories: anthropometry, metabolism, cardiovascular function, inflammation, hematology, liver function, kidney function, and physical function. Our catalog of SASP protein associations with human aging traits can serve as a first step to prioritizing SASP proteins and aging traits for the development of diagnostics and therapeutics for healthy human aging.

## Method

### Studies and Clinical Trait Measurements

This study used previously collected data from research participants in the Baltimore Longitudinal Study of Aging (BLSA)/GESTALT and the “Invecchiare in Chianti” (Aging in the Chianti Area, InCHIANTI) study. The range of participant ages was 22–93 years in BLSA/GESTALT and 21–102 years in InCHIANTI. The design of these studies and the methods used for clinical trait measurements are shown in Supplementary Material.

### Aptamer-Based Proteomics

For BLSA and InCHIANTI, proteomic profiles for 1 322 SOMAmers were assessed using the 1.3k SomaScan Assay at the Trans-NIH Center for Human Immunology, Autoimmunity, and Inflammation (CHI), National Institute of Allergy and Infectious Diseases, National Institutes of Health (Bethesda, MD), see Supplementary Material. The data reported are SOMAmer reagent abundance in relative fluorescence units (RFU), which represents a surrogate of protein concentration in the plasma sample.

### Statistical Association Analysis

Protein RFU values were natural log-transformed and outliers outside 4 standard deviations were removed. Linear regression models were used to identify protein associations with age and clinical traits. The estimated glomerular filtration rate (eGFR) was calculated using the CKD-EPI Creatinine equation (2021) (20). To determine whether white blood cell (WBC) count or SomaScan plate should be adjusted for, WBC or plate were tested for association with the first 2 principal components (PCs) estimated from the SomaScan data collected from BLSA participants. Plate was associated with PC1 and PC2, so Plate ID was included as an adjustment covariate. WBC was not associated with PC1 or PC2, so WBC was not included as an adjustment covariate.

Protein associations with age in BLSA and InCHIANTI were identified using the following linear regression model:


$$\begin{array}{l} SOMAmer\;=\;age\;+sex\;+eGFR\;+clinic\;ID\;+\;race\;\left(BLSA\;only\right)\;+Plate\;ID \end{array}$$

Fasting glucose, IL6, and CRP values were log transformed prior to regression analysis; all other clinical traits were not transformed. Protein associations with clinical traits in BLSA and InCHIANTI were identified using the following linear regression model:


$$\begin{array}{l} trait\;=\;SOMAmer+age\;+sex\;+eGFR\;+clinic\;ID\;+race\left(BLSA\;only\right)\;+Plate\;ID \end{array}$$

All InCHIANTI participants were the same race, so race was not adjusted for in analyses in the InCHIANTI study. Clinic ID and Plate ID were coded as factors.

Eight of the SASP proteins interrogated by the SomaScan panel were assayed by more than one SOMAmer. To generate a single association result in each study for proteins assayed by multiple SOMAmers, the age or trait associations for the SOMAmers for each protein were combined using inverse-variance weighted fixed-effect meta-analysis. Once protein-level associations were estimated in BLSA and InCHIANTI, the association results from the 2 studies were combined using inverse-variance weighted fixed-effect meta-analysis.

**Figure 1.**
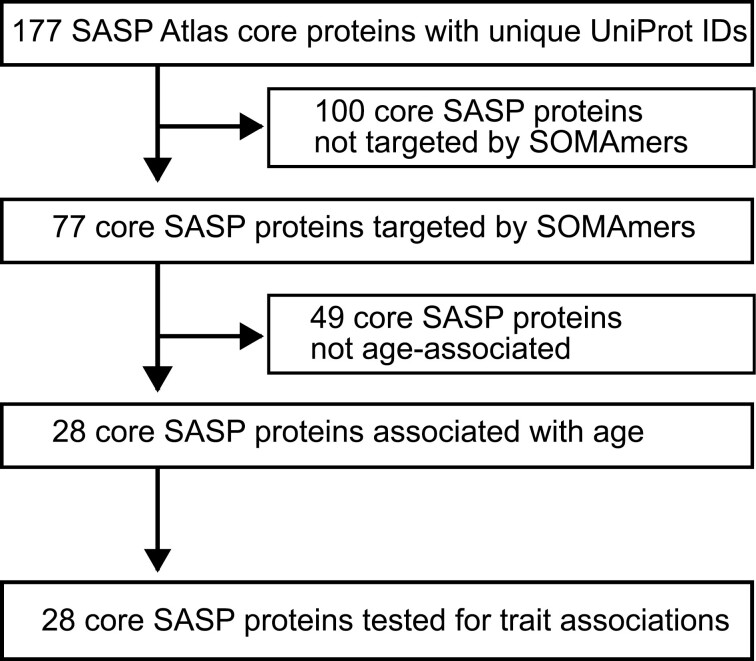
Selection of age-associated SASP protein for trait association analysis.

After the meta-analysis of BLSA and InCHIANTI results, the evaluation of significance proceeded in 2 stages (Figure 1). The first stage tested age associations with all 77 SASP proteins assayed by SOMAmers, and a Bonferroni correction was applied, resulting in a *p* value significance threshold of 6.5 × 10^−4^, that is, 0.05/77. A SASP was deemed to be significantly associated with age if the meta-analysis *p* value was less than or equal to the Bonferroni significance threshold. The second stage tested only the 28 age-associated SASP proteins for clinical trait associations, and a Bonferroni correction was applied for the number of age-associated SASP proteins, resulting in a *p* value significance threshold for trait associations of 0.0018, that is, 0.05/28 (Figure 1).

Heatmap visualization of SASP trait associations was created by performing complete hierarchical clustering of the Euclidean distance matrix of meta-analysis *Z*-statistics from the combined BLSA and InCHIANTI results, followed by plotting as a heatmap.

### Over-Representation Analysis

Over-representation analysis (ORA) was used to compare 2 proportions for each of the 12 clinical traits: (1) the proportion of significant trait associations among the age-associated SASP proteins, and (2) the proportion of significant trait associations among the proteins present on the SomaScan array, excluding the SASP proteins associated with age (non-SASP). To calculate proportions, significant trait associations were counted as associations with an unadjusted *p* value of ≤ .05. Proportions of significant associations were examined for each trait separately. To compare trait association proportions, a 2-sample conditional Fisher’s exact test was performed using the R function exact2×2::exact2×2, where the odds ratio (OR) represents the effect estimate (21). ORs greater than 1 indicate that the frequency of trait associations among SASP proteins is greater than what is found among non-SASP proteins. Significant enrichment or depletion was assessed using a 2-sided *p* value of .05.

## Results

Proteomic data from SomaScan arrays were obtained from 240 participants from the BLSA and 961 participants from the InCHIANTI study (total *N* = 1 201). Participants of BLSA and InCHIANTI included men and women. The mean age of InCHIANTI participants was higher than BLSA participants (Table 1). The range of participant ages was 22–93 years in BLSA and 21–102 years in InCHIANTI. All participants of the InCHIANTI study were of European ancestry. Of the BLSA participants, 168 (70%) self-reported as White, 54 (22.5%) as Black, and 18 (7.5%) as other (Chinese, Filipino, Japanese, Hawaiian, other Asian, or Other Pacific Islander, American Indian or Alaska Native, not classifiable, or other non-White). The clinical traits tested for SASP protein associations were grouped in the following categories that reflect multiple aspects of aging physiology and function: anthropometry (waist circumference), metabolism (glucose), cardiovascular (systolic and diastolic blood pressure [BP]), inflammation (C-reactive protein [CRP] and interleukin-6 [IL6]), hematology (red blood cell distribution width [RDW]), liver function (alkaline phosphatase), kidney function (blood urea nitrogen [BUN] and albumin), and physical function (grip strength and 6 m gait speed). The subset of BLSA participants selected for proteomic assessment was selected to be free of major age-related diseases to represent a healthy aging subsample; thus, it was unsurprising that age-related health risk factors were significantly different between participants of the 2 studies (Table 1).

**Table 1.** Characteristics of Study Participants

	BLSA		InCHIANTI		
Trait (unit)	*n*	Mean ± *SD* or %	*n*	Mean ± *SD* or %	*p* Value
Age (y)	240	57.5 ± 19.8	963	66.2 ± 15.2	<.001
Sex (% women)	240	50%	963	56%	<.001
Waist circumference (cm)	240	87.1 ± 12.0	947	91.2 ± 10.8	<.001
BP, systolic (mmHg)	240	113.5 ± 13.8	963	144.2 ± 21.5	<.001
BP, diastolic (mmHg)	240	68.1 ± 8.8	963	82.5 ± 9.4	<.001
Glucose (mg/dL)	236	87.4 ± 8.7	961	93.6 ± 23.8	<.001
IL6 (pg/ml)	240	3.7 ± 2.8	961	1.8 ± 3.2	<.001
CRP (μg/ml)	238	2.1 ± 2.8	949	4.0 ± 6.6	<.001
RDW (%)	239	13.3 ± 0.8	955	13.5 ± 0.9	<.001
BUN (mg/dl)	240	15.7 ± 4.5	961	33.1 ± 8.8	<.001
Alkaline phosphatase (units/L)	240	70.4 ± 17.9	961	200.2 ± 98.2	<.001
Albumin (g/dl)	240	3.9 ± 0.3	961	4.3 ± 0.3	<.001
Gait speed (m/s)	234	1.3 ± 0.2	941	1.1 ± 0.3	<.001
Grip strength (kg)	240	35.3 ± 12.0	836	31.9 ± 13.9	<.001

*Notes*: BLSA = Baltimore Longitudinal Study of Aging; BUN = blood urea nitrogen; CRP = C-reactive protein; IL6 = Interleukin-6; RDW = red cell distribution width.

*p* Value based on comparison of values between BLSA and InCHIANTI using 2-sample, 2-sided *t* test for continuous traits and χ^2^ test for categorical traits. *t* Test was performed using log-transformed glucose, IL6, and CRP values.

The 1.3k SomaScan panel contained many of the core SASP Atlas proteins induced by all 3 senescence inducers (irradiation, RAS, and ATV) reported in Basisty et al. (13). There were 177 unique UniProt identifiers from the published core SASP proteins (13), and 77 of the core SASP were targeted by SOMAmers (Supplementary Table 1). Although only 44% of the core SASP Atlas proteins were present on the SomaScan panel, the 77 SASP proteins on SomaScan were enriched for many of the same pathways as the full 177 core SASP proteins (Supplementary Table 2). Of the top 25% of the significantly enriched pathways for the core SASP, 81% of those pathways were significantly enriched (*Q* value ≤ .05) and 94% were nominally enriched (*p* value ≤ .05) by the SASP on the SomaScan panel (Supplementary Table 2).

**Figure 2.**
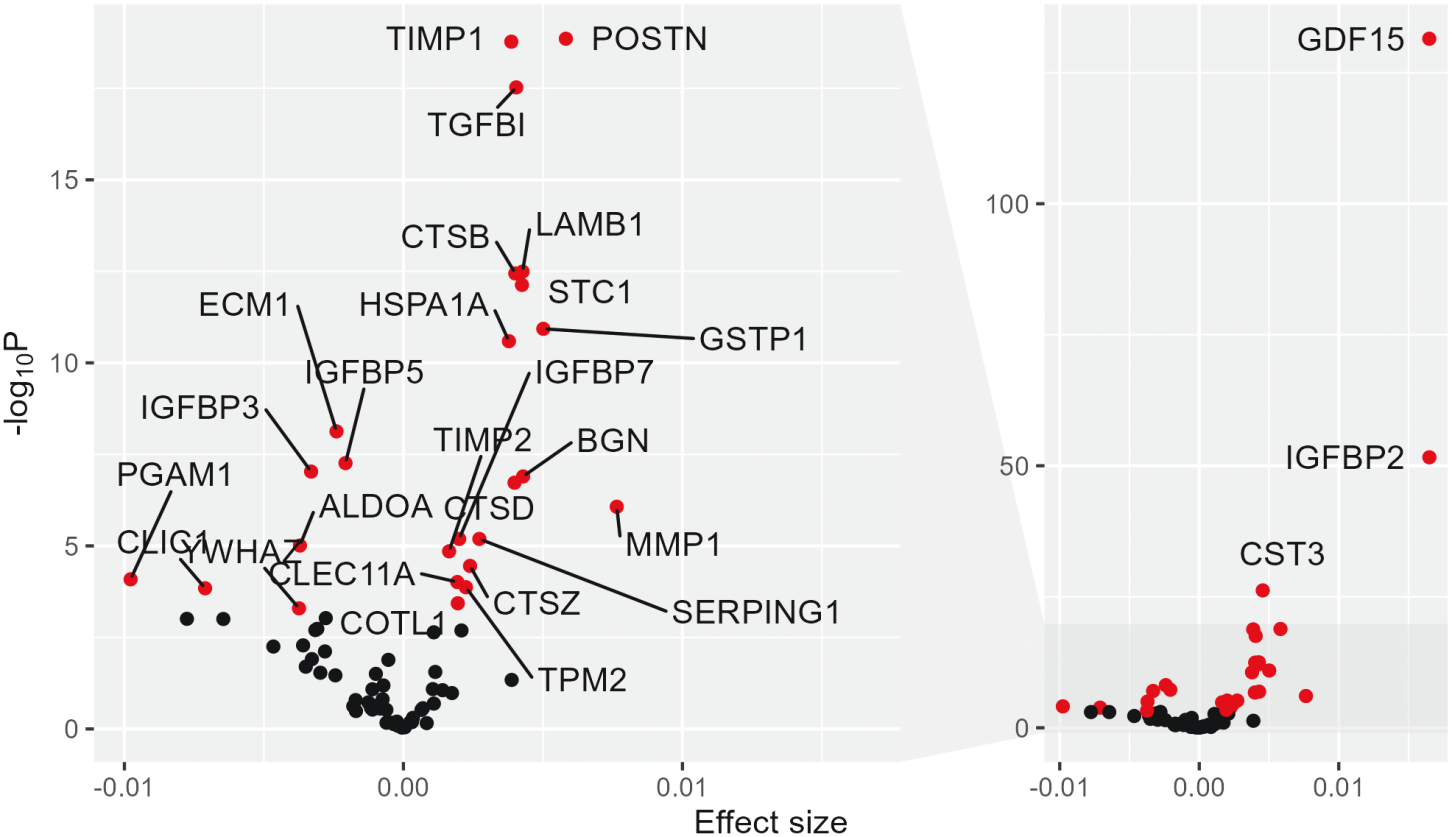
Volcano plot of SASP age associations. Effect size of age association on the x-axis, and significance of the age association represented as the -log_10_  *p* value of the age association on the y-axis. Full plot of the right-side labeling top 3 SASP age associations, and zoomed plot of lower segment of y-axis on the right side. Black points = not significantly associated after multiple testing with age. Red points = significantly associated after multiple testing with age. Gene names label red points.

SASP proteins were first tested for association with age, and only age-associated SASP proteins were subsequently tested for association with traits (Figure 1). Protein associations with age were identified in BLSA and InCHIANTI, and the results were combined in a fixed-effect meta-analysis. Of the 77 SASP proteins captured on the SomaScan array, 28 were significantly associated with age after Bonferroni correction in the meta-analysis (Figure 2, Supplementary Table 3). Age was associated with higher levels of 21 of the 28 age-associated SASP proteins, consistent with an age-associated increase in senescence burden and the accompanying SASP (Supplementary Table 3). The SASP proteins most significantly associated with age were GDF-15 (*P* = 3.3 × 10^−132^), IGFBP-2 (*P* = 2.4 × 10^−52^), and Cystatin-C (*P* = 6.1 × 10^−27^; Figure 2, Supplementary Table 3).

The 28 age-associated SASP proteins were tested for associations with 12 clinical traits in BLSA and InCHIANTI (Table 1), and results were combined in a fixed-effect meta-analysis. After multiple test corrections, 18 of the 28 age-associated SASP proteins were significantly associated with one or more of the clinical traits (Table 2, Supplementary Table 4). The pattern of trait associations across the 28 age-associated SASP proteins was visualized with hierarchical clustering of the trait association meta-analysis *z* scores (Supplementary Figure 1). A cluster was composed of GDF-15 and IGFBP-2, and these 2 proteins were significantly associated with age, inflammatory markers, fasting glucose, RDW, and grip strength (Supplementary Figure 1, Table 2).

**Table 2.** Number of Traits Significantly Associated with Each Age-associated SASP

Gene symbol^*^	Number significant^**^	Traits associated^***^	Proportion significant
IGFBP2	8	Waist circumference, glucose, CRP, diastolic BP, albumin, RDW, grip strength, systolic BP	0.67
GDF15	7	Gait speed, grip strength, BUN, IL6, glucose, albumin, RDW	0.58
CST3	5	Gait speed, IL6, albumin, grip strength, BUN	0.42
SERPING1	3	Waist circumference, CRP, IL6	0.25
YWHAZ	3	Gait speed, waist circumference, grip strength	0.25
TIMP1	3	RDW, IL6, gait speed	0.25
CTSZ	3	Waist circumference, CRP, gait speed	0.25
LAMB1	2	Diastolic BP, waist circumference	0.17
POSTN	2	Gait speed, waist circumference	0.17
TIMP2	2	CRP, waist circumference	0.17
STC1	2	BUN, IL6	0.17
ALDOA	2	Gait speed, IL6	0.17
BGN	1	Glucose	0.08
CLEC11A	1	RDW	0.08
CLIC1	1	Gait speed	0.08
ECM1	1	CRP	0.08
IGFBP7	1	Albumin	0.08
PGAM1	1	Gait speed	0.08

*Notes*: BP = blood pressure; BUN = blood urea nitrogen; CRP = C-reactive protein; IL6 = Interleukin-6; RDW = red cell distribution width.

^*^Includes only age-associated SASP with at least 1 significant trait association.

^**^Significant after Bonferroni correction.

^***^Traits listed in order of increasing trait association *p* value.

Twelve of the age-associated SASP proteins were associated with 2 or more traits, and 7 were associated with 3 or more traits (Table 2). The age-associated SASP proteins associated with the highest number of traits after multiple test corrections were IGFBP-2, GDF-15, and Cystatin-C. IGFBP-2 was associated with the most traits (8 traits), followed by GDF-15 (7 traits), and Cystatin-C (5 traits; Table 2). Levels of all 3 proteins increased with age (Figure 2).

Examining the 3 most significant age-associated SASP proteins individually, higher levels of GDF-15 and Cystatin-C were associated in the direction of adverse health across a wide range of physiological features (Supplementary Figure 2A and B). Higher levels of GDF-15 were significantly associated with higher IL6 levels indicating higher inflammation levels, lower albumin levels, and higher BUN measurements consistent with worse renal function, higher fasting glucose measurements that can be a sign of metabolic dysregulation, higher RDW which is a hematological biomarker associated with mortality risk, and poorer physical performance (lower grip strength and slower gait speed; Supplementary Figure 2A). Cystatin-C shared the same associations as GDF-15, with the exception of fasting glucose and RDW (Supplementary Figure 2B). IGFBP-2 displayed a more complex profile of associations, in which higher levels of IGFBP-2 were associated with adverse trait profiles (lower albumin, lower grip strength, and higher RDW) and favorable trait profiles (lower BP, CRP, glucose, and waist circumference; Supplementary Figure 2C).

Eight SASP proteins were targeted by more than 1 SOMAmer, represented by the following gene symbols: *C3*, *FN1*, *HSP90AB1*, *YWHAQ*, *TWHAB*, *YWHAE*, *YWHAZ*, and *CLEC11A* (Supplementary Table 5). Of these 8 proteins, only 2 (gene symbols *YWHAZ* and *CLEC11A*) were significantly associated with age and at least 1 other trait (Table 2). We assessed whether the SOMAmers that targeted the same protein were consistently associated with the traits shown in Table 2 for *YWHAZ* and *CLEC11A* gene products. The *CLEC11A* gene product was targeted by 2 SOMAmers (SL004362 and SL004363), and the associations with age and RDW were in the same direction for both SOMAmers in BLSA and InCHIANTI (Supplementary Table 5). The *YWHAZ* gene product was also targeted by 2 SOMAmers: SL017611 and SL005688 (Supplementary Table 5). SL017611 targeted 7 proteins, all of which are part of the 14-3-3 protein family. For grip strength, waist circumference, and gait speed, associations were in the same direction for both SOMAmers (SL017611 and SL005688) in BLSA and InCHIANTI. For age, the only discordant association was with SL017611 in BLSA (Supplementary Table 5). For the SOMAmers targeting the same protein, SOMAmer results were meta-analyzed within each study, and the summary protein result was then meta-analyzed between the 2 studies (see earlier section “Method”).

Age-associated SASP proteins were enriched for associations with waist circumference, RDW, fasting glucose, and IL6 (Table 3). Traits relevant to metabolism (waist circumference, fasting glucose), inflammation (IL-6), and hematology (RDW) could be more likely to be influenced by SASP, but our association study does not provide evidence for specific mechanisms that could link SASP to these traits. There were 5 or more Bonferroni-corrected significant SASP protein associations with gait speed, waist circumference, IL6, and CRP (Table 3). Grip strength, RDW, and albumin were each associated with 4 SASP proteins after multiple test corrections (Table 3).

**Table 3.** Traits Enriched for SASP Associations

Trait	Number significant^*^	Gene symbols of proteins significantly associated^**^	Enrichment *p* Value^***^
Usual gait speed	9	GDF15, CST3, PGAM1, YWHAZ, CLIC1, POSTN, ALDOA, CTSZ, TIMP1	0.17
Waist circumference	7	IGFBP2, TIMP2, CTSZ, SERPING1, POSTN, LAMB1, YWHAZ	0.0081
Log IL-6	6	GDF15, CST3, STC1, TIMP1, SERPING1, ALDOA	0.0014
Log CRP	5	IGFBP2, TIMP2, CSTZ, ECM1, SERPING1	0.51
Grip strength	4	GDF15, IGFBP2, CST3, YWHAZ	0.17
RBC distribution width	4	IGFBP2, TIMP1, CLEC11A, GDF15	0.010
Albumin	4	IGFBP2, IGFBP7, CST3, GDF15	0.076
Log glucose	3	IGFBP2, GDF15, BGN	0.020
Blood urea nitrogen	3	GDF15, STC1, CST3	0.19
Diastolic BP	2	IGFBP2, LAMB1	0.54
Systolic BP	1	IGFBP2	0.52

*Notes*: BP = blood pressure; CRP = C-reactive protein; IL6 = Interleukin-6; RBC = red blood cells.

^*^Bonferroni *p* value ≤ .05, of the 28 age-associated SASP proteins.

^**^Gene symbols listed in order of increasing trait association *p* value.

^***^Fisher’s exact test comparing the proportion of nominally significant SASP-trait associations among the 28 age-associated SASP proteins with the proportion of nominally significant trait associations among all 1 269 non-SASP proteins represented on SomaScan panel.

## Discussion

We present a catalog of human aging trait associations with a large number of SASP proteins in 2 clinical studies of human aging, 1 in the USA and 1 in Europe, capturing different human demographics. Among the 77 SASP proteins examined in 1 201 participants from 2 studies of human aging (BLSA/GESTALT and InCHIANTI), 28 were significantly associated with age after correction for multiple testing. Among the 28 age-associated SASP proteins, 18 were associated with 1 or more clinical age-related traits. GDF-15, Cystatin-C, and IGFBP-2 were the most significantly associated with age, and each of these proteins was associated with 5 or more clinical traits. GDF-15 and IGFBP-2 were both significantly associated with age, inflammatory markers, fasting glucose, RDW, albumin, and grip strength. Higher levels of Cystatin-C were significantly associated with higher IL6, higher BUN, lower albumin, and poorer physical performance (lower grip strength and slower gait speed), and higher levels of Cystatin-C’s target, Cathepsin Z, were significantly associated with higher waist circumference, higher CRP, and slower gait speed. In addition to the top 3 age-associated SASP proteins, there were 9 SASP proteins associated with 2 or more traits and 6 SASP proteins associated with 1 trait after multiple test corrections. Consistent with our findings that GDF-15, IGFBP-2, and Cathepsin Z were associated with multiple aging-related traits in our study, these proteins were among 44 proteins reported to be associated with age and mortality risk and were also reported to influence the association between *foxo3* genotype and reduced mortality in Americans of Japanese ancestry living in Hawaii (22). SASP proteins were enriched for trait associations with waist circumference, IL6, RDW, and fasting glucose, which prioritizes these traits for future examination of SASP protein associations. Our study lays the groundwork for future intervention studies to test the geroscience hypothesis by providing evidence that SASP proteins are associated with multiple aging traits, prioritizing which SASP proteins could be used as biomarkers, and prioritizing which traits should be examined in senolytic intervention studies.

In addition to identifying specific SASP proteins associated with aging traits in humans, our work also provides insight into traits that could be particularly influenced by SASP proteins. Our enrichment analysis determined that age-associated SASP proteins are enriched for associations with waist circumference, fasting glucose levels, IL6 levels, and RDW compared with non-SASP proteins. Although 9 SASP proteins were significantly associated with usual gait speed after multiple test corrections (Table 3), this trait was not enriched for SASP associations, indicating that a high proportion of proteins on the SomaScan panel were also associated with gait speed. By testing a wide range of clinical aging traits, our work prioritizes the investigation of metabolic measures, inflammatory markers, and RDW for future work investigating the role of SASP in human aging. There is ample evidence that waist circumference, fasting glucose levels, IL6 levels, and RDW are significantly associated with age-related outcomes and mortality in the older adults (23–26). The relationship between SASP proteins and metabolism is unsurprising, as a link between senescence and metabolic diseases has been recognized (27). There are certainly other traits relevant to healthy aging that should be examined in future studies, but based on our work, further examination of SASP protein associations with metabolic traits, inflammatory markers, and RDW appears to be warranted.

GDF-15 was the most significantly associated with age among the SASP proteins, and it was also associated with 7 traits related to physical function, kidney function, inflammation, metabolic function, and hematology. GDF-15 is a core member of the SASP, and our results add to the substantial body of work supporting GDF-15’s role in the development of aging traits in humans. GDF-15 has been found to be associated with mortality, incident cardiovascular events, inflammatory biomarkers, physical function, cortical bone density loss in women, metabolic markers, interstitial lung abnormalities, and kidney function (28–31). GDF-15 has also been associated with incident disability (32) and incident anemia (33). GDF-15 was associated with slower gait speed in a candidate protein study in BLSA (34). In a proteome-wide study of gait speed decline conducted in the Cardiovascular Health Study and the Framingham Heart Study, GDF-15 was the most significantly associated protein, which provides external confirmation of our results (35). Our findings combined with previous results provide strong evidence that GDF-15 is one of the most clinically relevant SASP factors.

The second most significant age-associated SASP protein was Cystatin-C, and it was also associated with gait speed, IL6, serum albumin, grip strength, and BUN. Cystatin-C is a cysteine protease inhibitor produced at a constant rate by most cells, is freely filtered in the glomeruli, and is a sensitive marker of renal function that is commonly used to calculate eGFR (20,36,37). Our observed associations with Cystatin-C were adjusted for eGFR based on serum creatinine. The CST3 gene product, Cystatin-C, has, however, been shown to add to creatinine’s estimate of renal function and Cystatin-C strengthens the association of eGFR with end-stage renal disease and mortality (37). Thus, our observed Cystatin-C associations could reflect impaired renal function that was not fully adjusted for using creatinine-based eGFR. Alternatively, our observed associations could also reflect Cystatin-C’s biological role as a cysteine protease inhibitor. Cystatin-C is a member of the type II super-family of cystatins that inhibits cysteine proteases (cathepsins). Cystatin-C and its target cathepsins are involved in protein turnover, activation of precursor proteins, immune function (inflammation, inflammation-induced responses, and antigen presentation), and apoptosis (38). Indeed, Cystatin-C was associated with inflammation after adjustment for renal function in older adult participants (39). In addition, Cystatin-C’s inhibition of cathepsins has been implicated in the regulation of aging-related neurodegeneration (40). In our study, we cannot necessarily distinguish between Cystatin-C associations reflecting the incomplete adjustment for renal function or the biological role of Cystatin-C, but in support of our associations reflecting the biological role of Cystatin-C as a cathepsin protease inhibitor, we found that Cathepsin Z (gene name *CTSZ*), a SASP protein and Cystatin-C target, was also associated with many of the same outcomes as Cystatin-C. After multiple test correction, Cystatin-C and Cathepsin Z were significantly associated with slower gait speed and higher inflammation (IL6 for Cystatin-C and CRP for Cathepsin Z; Table 2). Future investigations will be needed to disentangle the multiple effects of Cystatin-C that could underlie SASP associations with aging.

Although higher levels of GDF-15 and Cystatin-C were associated with adverse health outcomes, IGFBP-2 showed a mixture of adverse and beneficial associations. Adverse IGFBP-2 associations were observed with reduced renal function (lower serum albumin), higher RDW, and lower grip strength (Supplementary Figure 2C). Potentially beneficial IGFBP-2 associations were observed with lower systolic and diastolic BP, lower fasting glucose, and lower waist circumference (Supplementary Figure 2C). IGFBP-2 is a member of the IGFBP family that regulates IGF signaling by controlling the distribution, function, and activity of IGF. IGFBP-2 has been shown to be associated with all-cause mortality (31), and insulin sensitivity modifies IGFBP-2’s association with mortality (41). In addition, IGFBP-2 was found to mediate the relationship between RDW and mortality (42). IGFBP-2 has also been shown to be associated with early stages of Alzheimer’s disease progression (43). Our findings of adverse and beneficial trait associations with IGFBP-2 reflect the complicated nature of SASP and cellular senescence that can have different context-dependent effects. It is not unprecedented for SASP to have beneficial effects (44). Cellular senescence is a driver of both physiologically beneficial phenotypes and age-related pathologies owing to the heterogeneous phenotypes of senescent cells that inhabit an organism or tissue, or “senotype” (45), which is dependent on biological context and environmental factors. For example, studies in mice have shown that senescent cells serve beneficial physiological roles such as wound healing (46), tumor suppression, restricting fibrosis in some contexts (47), and embryonic development (48). Further work is warranted to disentangle the multiple effects of IGFBP-2.

In addition to the top 3 age-associated SASP proteins, 15 age-associated SASP proteins were also associated with at least 1 trait (Table 2). Many of these proteins have been established to play important biological roles that affect aging. For example, tissue inhibitor of metalloproteinases 1 (TIMP-1), which we found to be associated with age, RDW, IL6, and gait speed, and tissue inhibitor of metalloproteinases 2 (TIMP-2), which we found to be associated with age, CRP, and waist circumference, are both inhibitors of matrix metalloproteinases (MMPs). The balance between TIMPs and MMPs affects extracellular matrix (ECM) and tissue remodeling and has been implicated in the etiology of several human diseases (49). Indeed, circulating TIMP-1 levels have been associated with femoral neck bone mineral density (BMD) loss and incident hip fracture in older men (50). Consistent with our finding that TIMP-1 is associated with gait speed, a recent proteome-wide association study of gait speed decline identified TIMP-1 as one of the most significant associations (35). Moreover, TIMP-2 was found to be necessary for hippocampal-dependent cognitive benefits to aged mice conferred by human umbilical cord plasma proteins (51). In addition to TIMP-1 and TIMP-2, Plasma protease C1 inhibitor (gene name *SERPING1*) that regulates complement activation was previously reported to increase with age (52), and was associated in our study with age, waist circumference, CRP, and IL6. These proteins and those shown in Table 2, Supplementary Table 3, and Supplementary Table 4 establish a catalog of SASP proteins to be pursued in further clinical research studies of aging.

Complementary studies of SASP protein associations with a selection of human aging traits using different panels of SASP proteins have been conducted. Consistent with our findings, Shin et al. reported that GDF-15 and IGFBP-2 were the most significantly age-associated SASP proteins (14). An index of SASP proteins was associated with antidepressant response to late-life depression in a nonrandomized trial of 416 older adult participants with major depressive disorder (16), but neither GDF-15 nor IGFBP-2 were included for consideration in the development of their SASP index. An examination of 24 circulating SASP proteins found significant associations with post-surgical outcomes and a frailty index built from comorbidity counts and activities of daily living among 115 biobank individuals, 97 older adults undergoing surgery for severe aortic stenosis, and a case-control study of older women undergoing ovarian cancer surgery (18 cases, 18 controls) (15). Even though there are likely key differences between the BLSA and InCHIANTI research participants from our study and individuals undergoing surgery from the Schafer et al. study, GDF-15 trait associations were found in both of our studies. Besides GDF-15, there were no other proteins in common between the SASP panel tested in Schafer et al. and the SASP atlas tested in this report. The distinct SASP components likely reflect the different approaches to identifying SASP proteins in humans. Our study using the SASP atlas is based on the SASP proteins identified using unbiased mass-spectrometry to detect proteins secreted by different cell types undergoing senescence from 3 senescence inducers (13), whereas the 24 SASP proteins from Schafer et al. were candidate proteins informed by model organism studies which were subsequently tested for senescence-induced secretion by irradiation in human cells (15). The 24-protein SASP panel from Schafer et al. was expanded to a panel of 27 proteins in a recent study of 1 377 LIFE study participants (17). Consistent with our findings, Fielding et al. reported that higher levels of GDF-15 were associated with older age and lower grip strength (17). Even with the expanded panel of 27 SASP proteins, only GDF-15 was in common with the SASP proteins examined in this report, presenting challenges to the comparison of results. Further complicating the comparison of results is the fact that our analysis adjusted for renal function, consistent with proteomic analysis conducted by Tanaka et al. (19) and Liu et al. (35), while renal function was not adjusted for in Schafer et al. (15) or Fielding et al. (17). As the field establishes and refines clinical studies of senescence and SASP on human aging, there is a need for consistent covariate adjustment and consistent sets of SASP proteins to establish a strong base of evidence regarding SASP effects on human aging.

There are limitations and strengths to this study. Limitations include the fact that not all core SASP proteins from the SASP atlas were assayed by the 1.3k SomaScan panel. Furthermore, senescent cells are not the only cell type that releases SASP proteins. With these limitations in mind, there are multiple strengths to this study. Strengths include our use of data from 2 cohort studies and the combination of results using meta-analysis. Significant associations from meta-analysis display consistency between studies. Furthermore, the human studies analyzed in this study did not contain individuals selected for particular diseases or conditions, making it more likely that our results generalize to the larger population of community-dwelling older adults. The subset of BLSA participants with proteomics data was selected to be free of disease and functional limitations, perhaps limiting the generalizability of our findings to the older adults without a major burden of disease. However, such strict selection criteria were not applied to participants from the InCHIANTI study, which should facilitate generalizability. Indeed, we have noted in our discussion that many of the proteins associated with aging traits in our study, for example, GDF-15, IGFBP-2, Cystatin-C, TIMP-1, TIMP-2, and Plasma protease C1 inhibitor, have also been previously reported to be associated with related traits. An additional strength of our study is that the aptamer proteomics platform we used captured more SASP proteins than other focused SASP studies to date. In addition, a broad range of traits was examined, providing insights into complex aspects of aging that encompass harmful and beneficial effects.

In conclusion, our results support the relevance of SASP proteins to human aging, identify specific traits that are potentially affected by SASP, and prioritize specific SASP proteins as biomarkers of human aging. As major efforts are being made to develop drugs that selectively eliminate senescent cells (senolytics) or suppress the SASP (senomorphics), prioritized biomarkers will be essential for tracking the efficacy of treatments during human trials. In this study, we have identified several SASP candidates that should be tracked in relation to specific clinical traits, for example, gait speed, “inflammaging,” and glucose metabolism, during senotherapeutic trials. Further replication of our findings in multiple study populations of older adult individuals would provide additional evidence supporting these associations as indicators of SASP activity relevant to human aging. Ideally, validation of our proposed SASP-associated markers will be carried out in senolytic trials. Future work could explore additional phenotypes of aging that were not examined here, such as cognitive function, disability, mortality, and other relevant outcomes that are monitored during drug trials.

## Supplementary Material

glad265_suppl_Supplementary_Tables_S1-S5

glad265_suppl_Supplementary_Figures_S1-S2
