# Supplementary material for: Proteomic Analysis of the Senescence-Associated Secretory Phenotype: GDF-15, IGFBP-2, and Cystatin-C Are Associated With Multiple Aging Traits
Source: J Gerontol A Biol Sci Med Sci. 2023 Nov 20;79(3):glad265. doi: 10.1093/gerona/glad265 (PMC10876076; doi:10.1093/gerona/glad265)

**Supplemental Material**

**Proteomic Analysis of the Senescence Associated Secretory Phenotype (SASP): GDF-15, IGFBP-2, and Cystatin-C Are Associated with Multiple Aging Traits**

eMethods. Supplemental methods.

eFigure 1. Heatmap of SASP trait associations.

eFigure 2. Trait associations of three SASP proteins with highest count of significant age associations.

Supplemental Tables found in tabs within Excel file.

eTable 1. SASP factors present on SomaScan v1.3.

eTable 2. Reactome pathways enriched by SASP and SASP on SomaScan.

eTable 3. SASP factors associated with age.

eTable 4. Clinical trait associations for age-related SASP factors.

eTable 5. SOMAmer age and trait associations for proteins targeted by multiple SOMAmers

**eMethods. Supplemental methods.**

**Clinical research study descriptions**

The BLSA started in 1958 as a population-based study that aimed to characterize physiological and functional aspects of aging (1). The GESTALT study began in 2015 and aimed to discover molecular biomarkers of ‘healthy’ aging. The GESTALT study protocols were similar to those from BLSA (2). Both BLSA and GESTALT recruited participants aged 20 years and older from the Washington DC/Baltimore metropolitan area (age range 22-93 years among participants with proteomics data). Study entry criteria for BLSA and GESTALT have been previously described, and include the absence of chronic disease, cognitive impairment, and functional impairment (2). The BLSA and GESTALT study protocols were approved by the Internal Review Board (IRB) of the National Institutes of Health and all participants provided written informed consent.

The InCHIANTI study is a population-based epidemiological study aimed at evaluating factors that influence mobility in the older population living in the Chianti region in Tuscany, Italy. Details of the study have been previously reported (3). Briefly, participants were selected from the population registry of Greve in Chianti (a rural area: 11,709 residents with 19.3% of the population older than 65 years of age) and Bagno a Ripoli (Antella village near Florence; 4704 inhabitants, with 20.3% older than 65 years of age). The age of study participants ranged between 21 and 102 years. The study protocol (exemption #11976) was approved by the Italian National Institute of Research and Care of Aging Institutional Review and approved by the IRB of the NIH. All participants provided written informed consent. Sociodemographic information (age, sex) was obtained during a structured interview.

**Clinical trait measurements**

For BLSA, blood tests were performed at a Clinical Laboratory Improvement Amendments (CLIA) certified clinical laboratory at Harbor Hospital, home of the National Institute of Aging (NIA) intramural research program clinical unit. White blood cell count and red blood cell distribution width was measured as part of the standard CBC using SYSMEX SE-2100 (Sysmex, Kobe, Japan). Serum creatinine was measured using isotope dilution mass spectrometry (IDMS)-traceable serum creatinine assay. Albumin was measured using dye binding BCG. Blood urea nitrogen (BUN) was measured with diazo coupling. Total cholesterol (TC), alkaline phosphatase, and creatinine were measured with enzymatic methods. High density lipoproteins (HDL) and low density lipoproteins (LDL) were measured with dextran sulfate-magnesium precipitation method. Triglycerides were measured with colorimetric methods, and glucose was measured with glucose oxidase using the VITROS System (Ortho Clinical Diagnostics, Raritan, NJ, USA). Serum inflammatory markers Interleukin 6 (IL6) (R&D System, Minneapolis, MN, USA) and C-Reactive Protein (CRP) (ALPCO, Salem, NH, USA) were measured with enzyme-linked immunosorbent assay (ELISA). Waist circumference, body mass index (BMI), and blood pressure were objectively assessed during a standard medical exam. Grip strength was measured three times on each hand, values were averaged for each hand, and the highest average value across both hands was used. Usual gait speed was measured in two trials of a 6 meter walk, and the faster time was used.

For InCHIANTI, sociodemographic information (age, sex) was obtained during a structured interview. Waist circumference was measured at the midpoint between the lower rib and iliac crest. Resting blood pressure was measured using a mercury sphygmomanometer. Usual pace walking speed was measured with a 4-meter trial. Hand grip strength was measured with a handheld dynamometer. Serum creatinine was measured using kinetic-calorimetric assay based on the Jaffe method (Baylor Diagnostics). Albumin was measured using agarose electrophoretic technique (Hydragel Protein (E) 15/30, Sebia Issy-les-Moulineaux, France), alkaline phosphatase was measured using automated colorimetric method (Roche Diagnostics, GmbH, Mannheim, Germany), CRP was measured with ELISA and calorimetric competitive immunoassay (Roche Diagnostics, GmbH, Mannheim, Germany), IL-6 was measured with Biosource CytoScreen ultrasensitive kits, and glucose was measured using an enzymatic assay. Blood urea nitrogen was measured with an UV kinetic test and a Roche analyzer (Roche Diagnostics). Whole blood white blood cell differential count and RDW was assessed using a Coulter Counter (LH 750 Hematology Autoanalyzer, Beckman Coulter Inc, Brea, CA).

**Aptamer-based Proteomics**

The 1.3k SomaScan Assay at the Trans-NIH Center for Human Immunology, Autoimmunity, and Inflammation (CHI), National Institute of Allergy and Infectious Diseases, National Institutes of Health (Bethesda, MD, USA) using the same methods as previously published (2,4). Out of the 1322 SOMAmer reagents, 12 hybridization controls, four viral proteins (HPV type 16, HPV type 18, isolate BEN, isolate LW123) and 5 SOMAmers that were reported to be non-specific (P05186; ALPL, P09871; C1S, Q14126; DSG2, Q93038; TNFRSF25, Q9NQC3; RTN4) were removed, thus leaving 1301 SOMAmer reagents in the final analysis. The experimental process for proteomic assessment and data normalization has been previously described (5,6). The data reported are SOMAmer reagent abundance in relative fluorescence units (RFU). The abundance of the SOMAmer reagent represents a surrogate of protein concentration in the plasma sample. Data normalization was conducted in three stages. First, hybridization control normalization removes individual sample variance on the basis of signaling differences between microarray or Agilent scanner. Second, median signal normalization removes inter-sample differences within a plate due to technical differences such as pipetting variation. Finally, calibration normalization removes variance across assay runs. Furthermore, there is an additional inter-plate normalization process that utilizes CHI calibrators designed to normalize across all experiments conducted at the CHI laboratory (5,6).

**References**

1. Shock NW, Greulick RC, Andres R, et al. Normal Human Aging: The Baltimore Study of Aging (NIH publication no.84-2450). www.grc.nia.nih.gov/blsahistory/blsa_1984/index.htm. Published 1984.

2. Tanaka T, Biancotto A, Moaddel R, et al. Plasma proteomic signature of age in healthy humans. *Aging Cell*. 2018;17(5):e12799. doi:10.1111/acel.12799.

3. Ferrucci L, Bandinelli S, Benvenuti E, et al. Subsystems contributing to the decline in ability to walk: bridging the gap between epidemiology and geriatric practice in the InCHIANTI study. *J Am Geriatr Soc*. 2000;48(12):1618-1625. doi:10.1111/j.1532-5415.2000.tb03873.x.

4. Tanaka T, Basisty N, Fantoni G, et al. Plasma proteomic biomarker signature of age predicts health and life span. *Elife*. 2020;9. doi:10.7554/eLife.61073.

5. Candia J, Cheung F, Kotliarov Y, et al. Assessment of Variability in the SOMAscan Assay. *Sci Rep*. 2017;7(1):14248. doi:10.1038/s41598-017-14755-5.

6. Cheung F, Fantoni G, Conner M, et al. Web Tool for Navigating and Plotting SomaLogic ADAT Files. *J Open Res Softw*. 2017;5. doi:10.5334/jors.166.

**eFigure 1. Heatmap of SASP trait associations.** Age and trait association Z-scores are displayed. Distance matrix uses euclidean distance, and complete hierarchical clustering was performed.


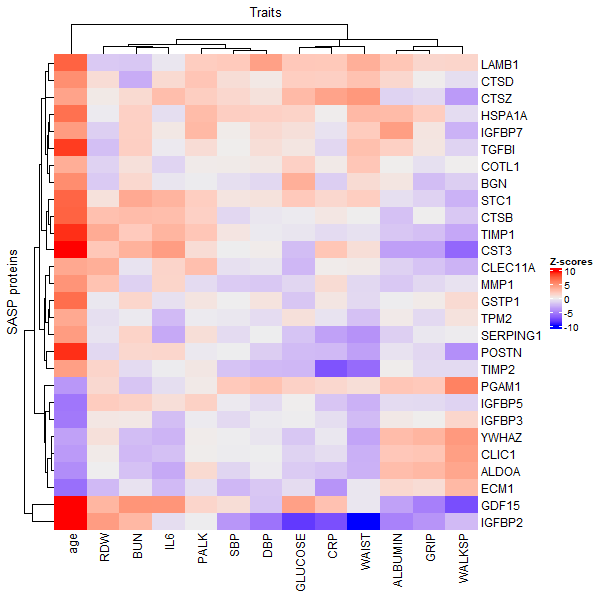


**eFigure 2. Trait associations of three SASP proteins with highest count of significant age associations.** A. GDF15. B. CST3. C. IGFBP2. Dashed lines mark a Z-score of zero, a null association. Dotted lines mark a Z-score corresponding to an unadjusted P-value 0.05. Red or green points indicate Bonferroni-significant associations that are detrimental or beneficial, respectively. Black point is age association, gray points are associations that are not Bonferroni-significant.


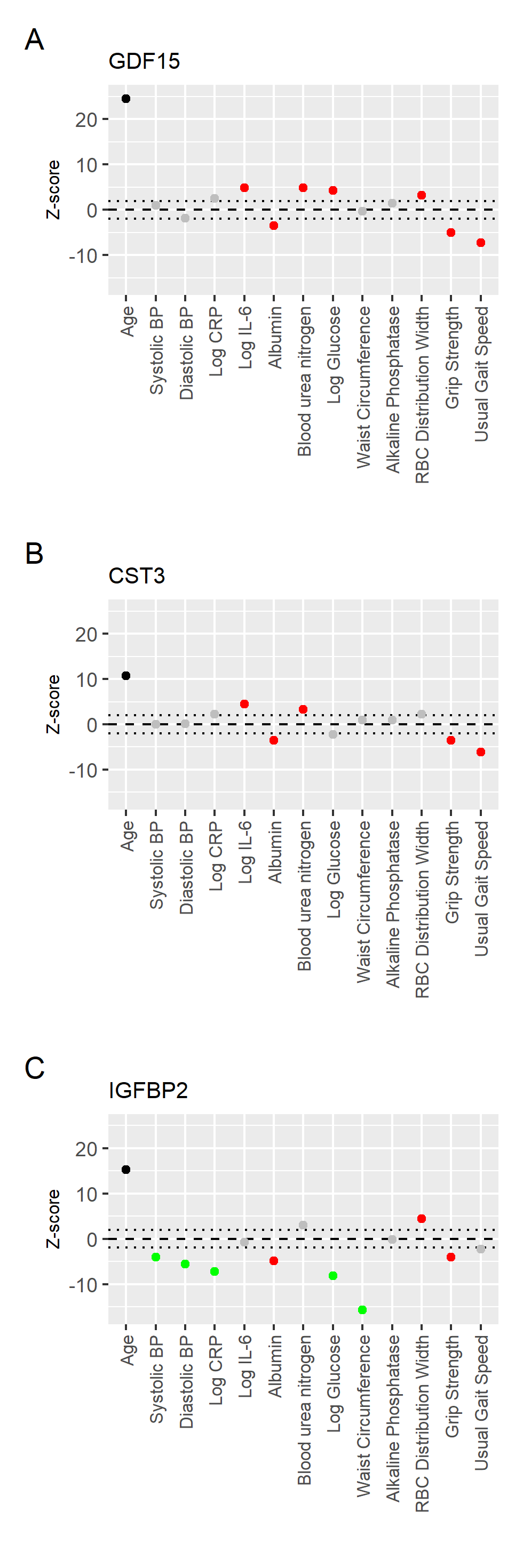

Supplement: glad265_suppl_Supplementary_Figures_S1-S2 [file glad265_suppl_supplementary_figures_s1-s2.docx]
